# Studying Force Patterns in an Alpine Ski Boot and Their Relation to Riding Styles and Falling Mechanisms

**DOI:** 10.3389/fspor.2021.557849

**Published:** 2021-04-13

**Authors:** Florian Nimmervoll, Umut Çakmak, Martin Reiter

**Affiliations:** ^1^Industrial Design Department, Institute of Space and Design, University of Art and Design Linz, Linz, Austria; ^2^Institute of Polymer Product Engineering, Johannes Kepler University Linz, Linz, Austria

**Keywords:** force patterns during skiing, skiing falling mechanisms, in lab and on the slope ski boot experiments, piezoresistive sensors, biomechanics

## Abstract

In skiing, performance and safety can depend on small details. Consequently, the measurement of forces within the ski boots, which represent the essential form-fitting and force transmitting interface during skiing, will lead to enhanced performance and more importantly safety. This study presents a methodology to measure force patterns (continuous data acquisition) under laboratory as well as realistic slope conditions. The force measurements will be analyzed to gain insights of the skiing style, skiing technique, specific falling mechanisms (i.e., boot induced anterior drawer, phantom foot, hyperextension of the knee joint, and valgus-external rotation). Furthermore, the locations of force sensors in a *overlap* designed ski boot are discussed in terms of practicability and applicability. These insights are of particular interest to derive release conditions for predictive binding systems and furthermore provide data to improve the style of skiing (e.g., turn release action or center of gravity behavior). For that purpose, a ski boot was instrumented with seven force (piezoresistive) sensors while the basic structure of the boot and the binding remained unchanged. Three sensors were placed on the insole to measure ground reaction forces as well as the contact forces between the skier's foot and the boot. The other four sensors were positioned at spoiler/shaft and toecap (front sole) regions of the ski boot. The locations of the force sensors within the ski-boot are defined with regard to the main body movement while skiing (body-related planes). In addition, a commercially available ski and body mount measuring system were utilized to correlate speed, inclination and body position with the force patterns occurring during skiing on the slope as well as simulating specific body positions on an inclined ramp under laboratory conditions. The measured force revealed that the toecap (upper) sensors provide insufficient even non-conclusive data to deduce significant patterns. However, the insole sensors (heel and front sole area) as well as the spoiler/shaft (back) sensors are more reliable and show characteristic patterns indicating forward or backward lean. These results will have an important impact to the development of predictiveelectro-mechanical bindings to prevent knee-related injuries, which, from a statistical point of view, concerns largely women and young athletes.

## 1. Introduction

The development of smart wearable electronics in the sports (Brunauer et al., 2020; Sperlich et al., 2020) and rehabilitation industry (Pȩdrys et al., 2020) leads the way to new market niches and even more it helps platform oriented business models to establish in conservative sports markets^1^. In recreational alpine skiing there are a number of wearables available (e.g., smartwatches, compact sensor units, smart insoles) to track and measure individual skiing performances as speed, stance, acceleration or vertical feet per ride. The miniaturization of sensing hardware, coupled with general acceptance of the smartphone as practical and ubiquitous control interface, makes a fast growing industry sector. Contrary to the fast development in wearable smart technologies, ski bindings remained mostly unchanged in their basic functional mechanics since the 1970s when safety release functions (side-wards release of the toe piece and upwards release of the heel unit; PTFE gliding plates) (Masia, 2018) have been introduced, which led to a decline in injury rates and a distribution shift from ankle and tibia fractures toward knee-related injuries (Kuriyama et al., 1980; Ettlinger et al., 1995; Johnson et al., 1997). The introduction of carving skis did not bring significant changes of knee injuries compared to other body parts (Burtscher et al., 2008). Ski safety bindings have not mitigated the high knee-related injury rates in a significant manner yet (Natri et al., 1999; Senner et al., 2014; Schulz, 2016), especially concerning female skiers (Kelsall and Finch, 1996; Burtscher et al., 2008; Ruedl, 2011; Sabeti, 2013; Brucker et al., 2014; Ruedl et al., 2014; Shea et al., 2014; Schulz, 2016). In 2003, 56% of injuries were located on the knee for women (Ruedl et al., 2009). The gender dependent hamstring to quadriceps ratio (HQ) is related to the tendency (susceptibility) to knee injury and is higher, the lower HQ is (Greenwald and Toelcke, 1997; EL Ashker et al., 2017). Lower Z values of the binding's release mechanism (i.e., determining the limiting torque), as recommended by the DIN-ISO 11088, may lead to lower injuries (Posch et al., 2017), however, that reasoning is controversial and neither applies to every skier nor riding style (e.g., aggressive riding, carving style, terrain, etc.). Also in youth and elite sports, severe knee injuries are common reasons for long term interruptions or discontinuations of athletic careers (Flørenes et al., 2009; Westin et al., 2012; Hildebrandt et al., 2017; Steidl-Müller et al., 2017). Especially, Koehle et al. (2002) addressed the ski binding design as one of the main preventative factors in alpine skiing. Within those knee-related falling and subsequent injury mechanisms concerning the human knee structure the *valgus-external rotation mechanism (VER)* as well as the *phantom foot mechanism* are the most common causes of structural overload (Ettlinger et al., 1995). Interestingly, Ruedl et al. (2009) stated a distribution change of anterior cruciate ligament (ACL) injury mechanisms from backward twisting fall (29%) toward forward twisting fall (51%)^2^ of female carving skiers. The related non-release (false negatives) of the ski bindings occurs 2.6 times more frequently than with males (Ruedl et al., 2011). Nonetheless, frequent binding release failure in backward twisting fall situations is still a well-known challenge in the development of new ski binding with improved safety capabilities (Ahlbäumer et al., 1999; St-Onge et al., 2004; Ruedl et al., 2009; Bere et al., 2011).

Research on the skier's body position, muscular stress and ground reaction forces (GRF) (Müller, 1994; Aune et al., 1995; Färber et al., 2018; Seifert et al., 2020) as well as studies on the influence of the boot's geometry (Benoit et al., 2005) and material properties (Petrone et al., 2013) can support product development, injury prevention and the development of skiing technique. Hereby, the utilization of pressure insoles is part of present day methods to measure forces within a ski boot or generally in orthopedics (Babiel et al., 1997; Stricker et al., 2010; Nakazato et al., 2011; Adelsberger, 2014). However, only GRF of the sole can be derived/measured with these pressure insoles.

Force distribution between the inner layer and the outer shell of a modern ski boot will provide insights about bio-mechanics, skiing technique as well as force patterns occurring during athletic skiing and in critical falling situations. Consequently, a measurement system with minimal effect and disturbance to both the ski boots' structure and the standing position of the skier was developed, DAQ programmed and built. In the research of Nakazato et al. (2011), Kistler force plates (three piezoelectric sensors) where mounted below the heel piece and the toe piece of an Atomic Race 1018 binding (Atomic Austria GmbH, Altenmarkt im Pongau, Austria). Force plates lead to a higher standing position and add a total weight of 4 kg on the skis by the amplifiers, controllers, and battery packs. Additionally, Nakazato et al. (2011) used a Pedar (Novel, Munich, Germany) pressure insole (PI) instead of the regular insole. It was suggested to use force plates only when 3D force data and torques are needed. For high dynamic situations, such as powder skiing or skiing moguls, Nakazato et al. (2011) concluded that PI measurements are preferable. Stricker et al. (2010) consider PI measurements, which represent 1D pressure forces, inaccurate for most bio-mechanical analyses in skiing and snowboarding, despite the high similarity to time-dependent force characteristics of skiing. After all, the reaction force is dependent on the material's mechanical behavior (constitutive behavior) and the geometry (structure) itself. Martínez et al. (2020) showed that interchangeability of sensor technology is not given.

Consequently, the hypothesis arises that all force measuring systems for athletes' locomotion are based on the foot sole only. We withdraw this hypothesis with our applied research efforts by selecting, characterizing mechanically as well as environmentally and programming flexible (piezoresistive) force sensors. Our findings are the first attempt to incorporate these types of sensors in ski boots with very promising outcomes. With this measurement system, the most important locations within the boot for force measurement (and monitoring) can be derived in order to correlate skiers' body position with ski boot forces. Most endeavors on developing electro-mechanical binding solutions have their origin in the search for a more reliable release in conjunction with knee injuries (Hull and Allen, 1981; Eseltine and Hull, 1991; Hull et al., 1997; Gulick and Mote, 2000; Senner et al., 2013). Even mechanically advanced binding solutions with multi-directional toe units can lead to dangerous accidental release and unintended operation (Ahlbäumer et al., 1999).

The objective of this study is to gain deeper insights into GRF (on the insole) in relation to forces within the ski boot itself to recognize specific force patterns. Therefore, we examine the duration and intensity of specific positions and movements under laboratory conditions as well as on the ski slope.

## 2. Materials and Methods

Considering the discussed researches and findings (see section 1), we identified a lack of measurement systems for descriptive analysis of boot-foot interactions occurring during skiing, respectively, in falling situations. Low interference of the measurement system on the confined foot within the boot (i.e., contact, interface and friction under thermal as well as mechanical loading) is required and is of tremendous importance. The sensor positions were chosen with respect to the skier movement and keeping in mind future tests with sensors integrated in injection molded plastic boots (tooling of the cavity has to be considered).

### 2.1. Ski Boot Instrumentation and Setup

**Figure 1:**
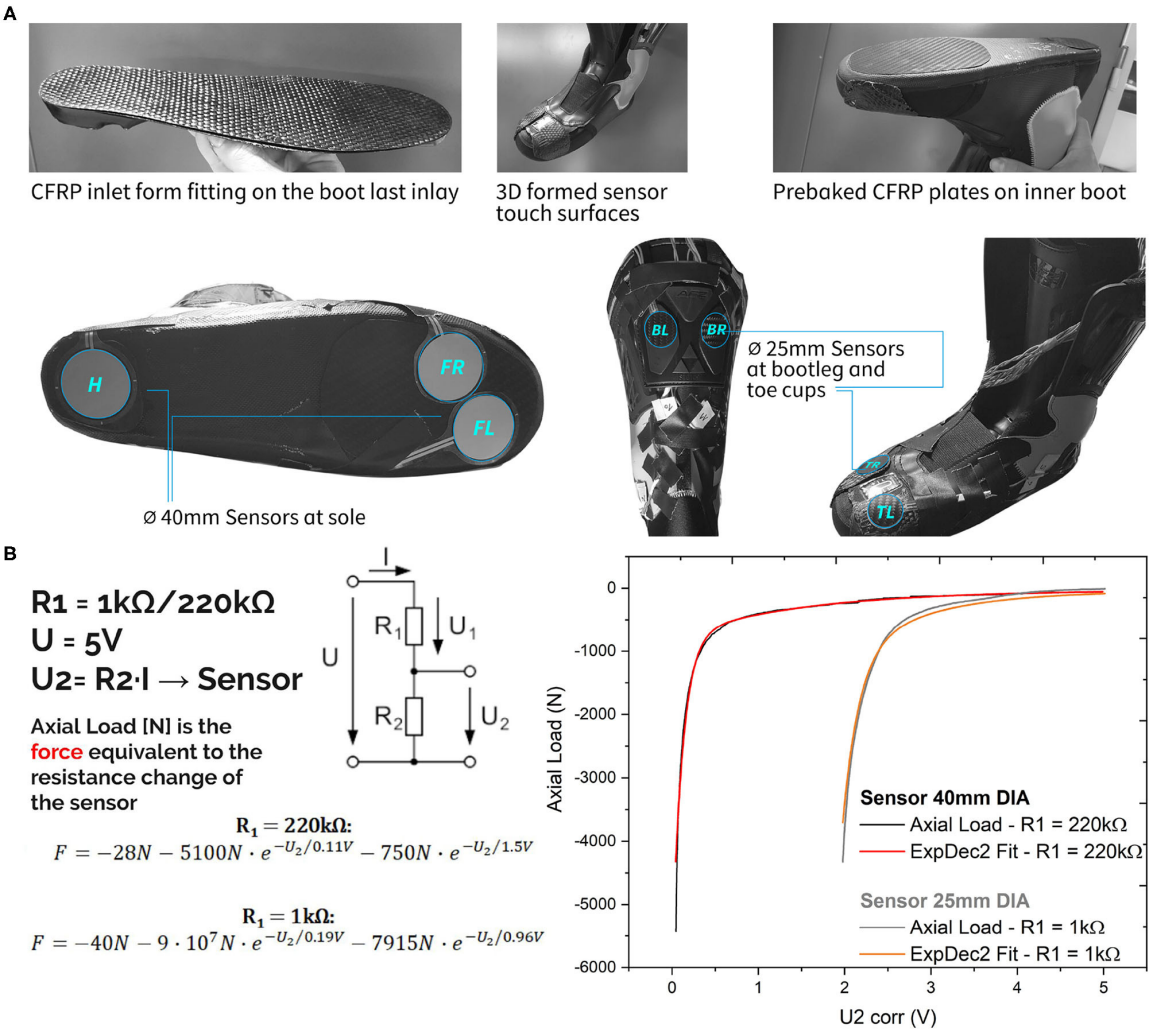
**(A)** Sensor positioning and carbon fiber reinforced polymer plates to improve force transmission. **(B)** Sensor calibration scheme.

A **right** Fischer ski boot (RC4 Curv Size 28.5 (Flex 130), Fischer Sports GmbH, Ried im Innkreis, Austria) has been modified to measure forces at seven locations within the boot. Two piezoresistive *Tactilus Sensors* (Sensor Products INC., NJ, USA) with a circular surface of 12.56 cm^2^ and 0.25 mm thickness have been put to a tailored carbon sole inlay. For better force transmission form-fitting carbon fiber laminated plates have also been fixed on the inner boot. Four round sensors with a surface area of 4.9 cm^2^ have additionally been placed at the ski boot's cuff and above the toes region as shown in Figure 1A. The positioning of these sensors was especially intended to measure force patterns of balanced skiing movements but also skiing when leaning forwards and backwards. Skis used for testing were Atomic Vantage 90 TI [2018/19 model, Allmountain Rocker, length = 184 cm, radius = 19.5 m, side cut = (129/90/119.5) mm].

#### 2.1.1. Sensor Type and Calibration

**Figure 2:**
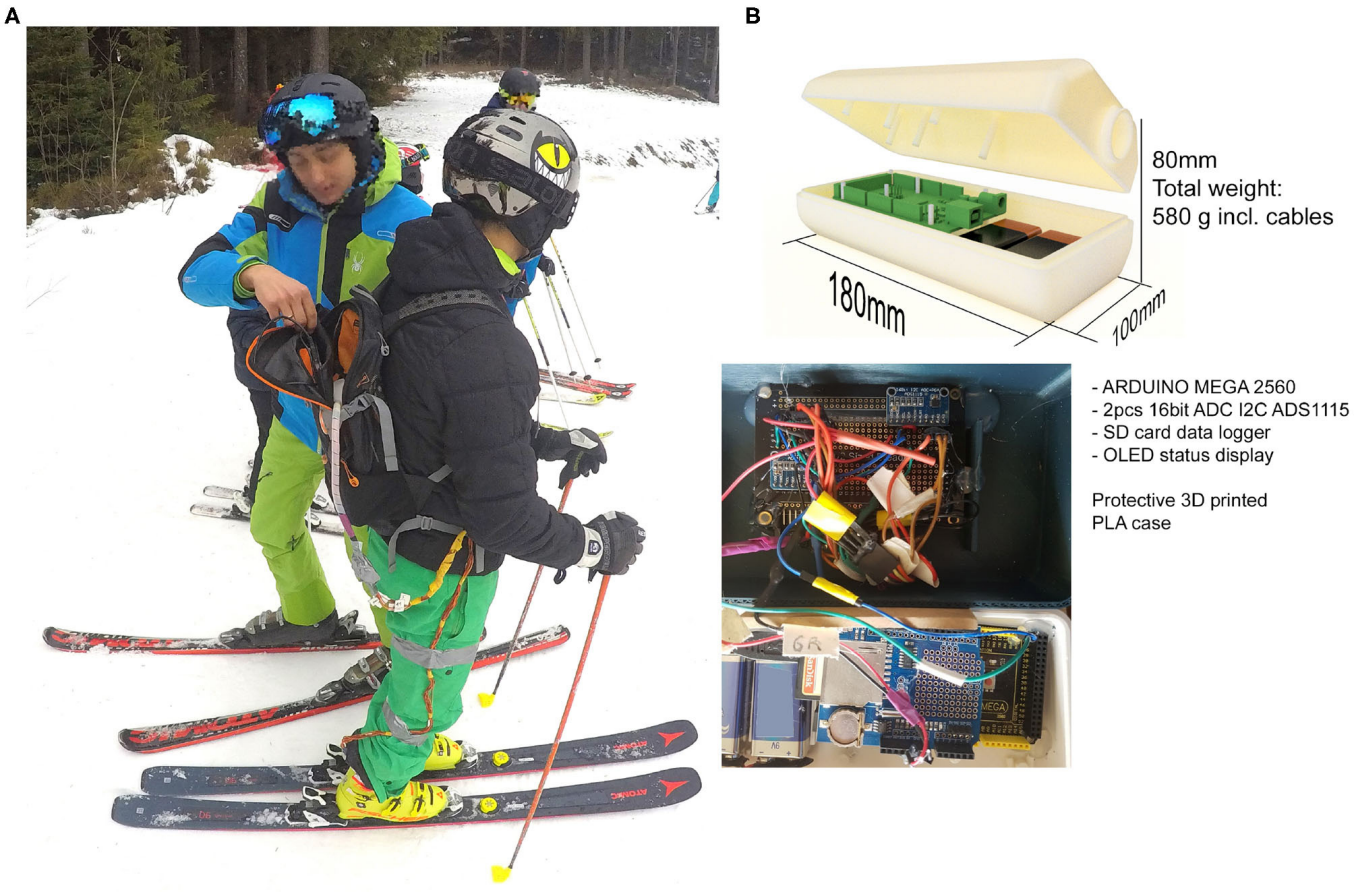
**(A)** Data acquisition system in a small backpack. **(B)** 3D-printed case of the DAQ system including the wiring of the sensor.

To achieve a light and mobile data acquisition (DAQ) setup the authors utilized a micro controller Arduino (MEGA 2560) assembled with two *ADS1115 ADC 4 Channel 16Bit I2C* analog to digital converters (see Figure 2B). Seven sensors were wired to the Arduino unit which is carried in the backpack. The sampling frequency of the system was at 100 Hz.

A mechanical test system MTS 852 Damper Test System (MTS, Minneapolis, MN, USA) was utilized to aggregate data for the calibration curves. Therefore, a 25 kN load cell has been used for the calibration in a compression loading configuration. The sensor has been put between two elastomeric foam sheets to ensure a soft contact of the metallic compression plate fixtures and hence a better force control during loading. The sensor was wired to a serial circuit including resistances of 1 and 220 kΩ. The applied total voltage U has been 5 V. The change of the sensor voltage U2 (see Figure 1B) has been recorded with the DAQ system of MTS while loading up to 5 kN. The resulting characteristic of the force F(U2) has been curve fitted by an exponential decay function as shown in Figure 1B. This calibration function has been implemented in the Arduino code to record the force values for the seven sensors during labor as well as field tests on the piste. Figure 2 shows the setup with the data capturing unit (580 g) placed in a backpack.

### 2.2. Testing the Boot-Sensor Setup Under Laboratory Conditions

**Figure 3:**
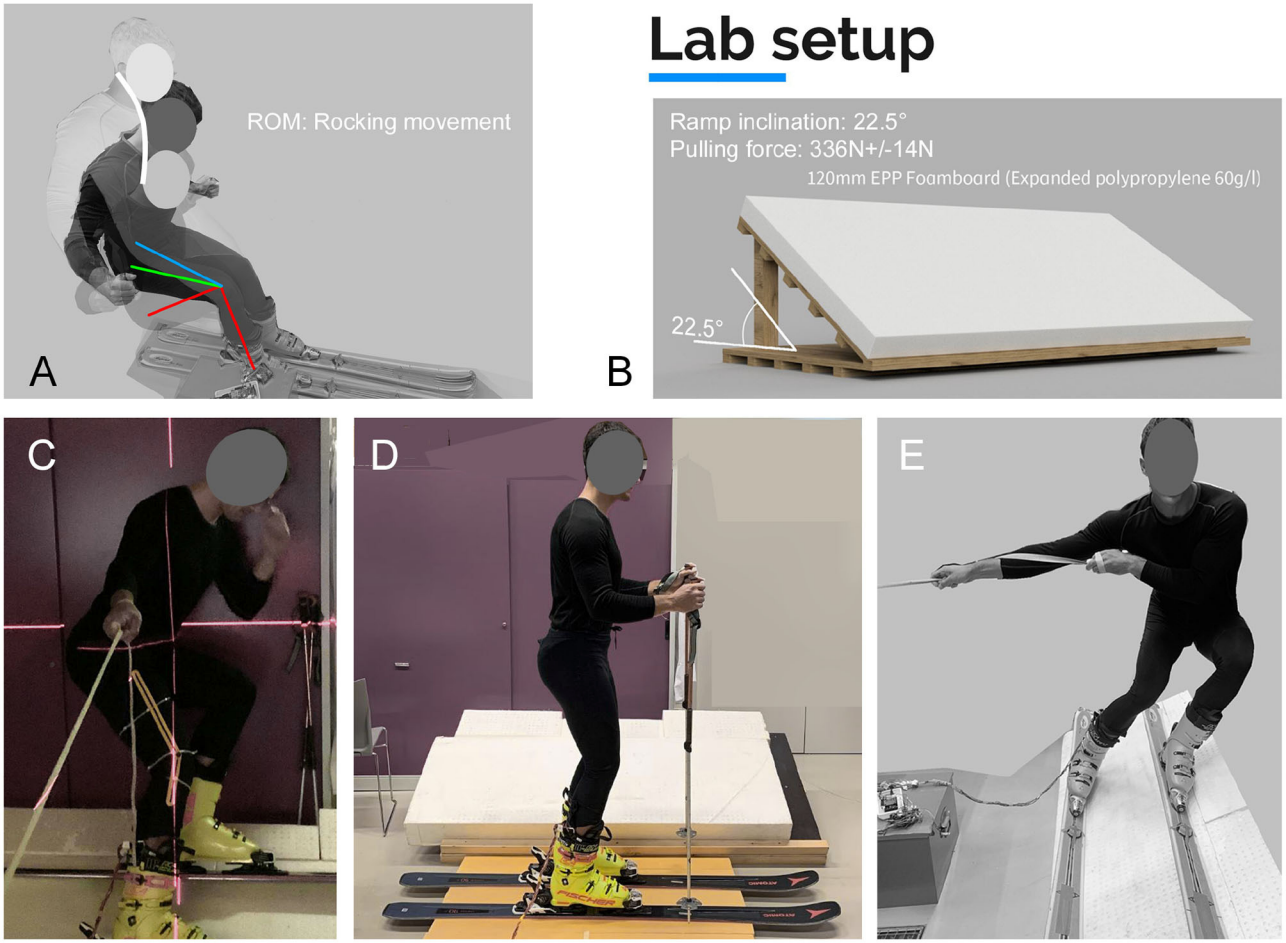
Lab experimental setup: **(A)** ROM at lay back movement, **(B)** Ramp setup, **(C)** Measuring of body angles using laser level and protractor. **(D)** Tare position, **(E)** Body position while pulling up the resistance weight in central stance.

An 22.5° inclined mobile ramp was covered by an expanded polypropylene foam (EPP 60 g/l) board. The foam board helped to gain edge grip. Hysteresis, respectively compression, felt natural even when applying high pulling forces up to 400 N at 22.5° slope angle (as illustrated in Figure 3B). As the foam structure received almost no structural damage, high reproducibility was given and the EPP gave a softer snow-like feeling to the participant. To obtain enough lateral force a rope attached to a 32 kg dead-weight at hip-level height was utilized, perpendicular to the ski direction. Resulting in pulling forces of 336 ± 14 N recorded with an electronic scale during the ramp testing procedure (ramp weight transfer).

#### 2.2.1. General Testing Sequence

The aforementioned calibration procedure started prior to every test including the flat land and ramp weight transfer tests followed by the sequences listed in Table 1. The injury-related body positions (IRBP) were simulated by getting into the specific position and altering the loading to see whether significant force peaks and/or patterns can be observed or not.

**Table 1:** Testing sequence after level calibration.

**Rocking motion level ground**	**Ramp pulling 336N**	**IRBP (see Figure 4)**
Central stance	Central stance (Figure 3E)	BIAD (Figure 4A)
Lay back	Lay back	Slip-catch (flat)
Forward lean	Forward lean	Phantom foot (flat, ramp) (Figures 4B,C)
		Inside edge (flat) (Figure 4D)

***Testing sequence*** Participants were informed of the potential risks and benefits of participation in this study prior to consenting to participate. This study was approved by the Ethics-Committee of the Federal State Upper Austria. Before starting the initial tests the participant (m = 90 kg; L = 189 cm) performed a 5 min warm-up including active movement and stretching of hamstrings and engaging hip mobility. Each test sequence started level ground where the system was calibrated (tared). The participant felt his center of gravity in the metatarsus area with a body position of slightly bend knees induced by the ski-boot geometry and an upper body forward lean, in which the poles did not touch the ground (see Figure 3E). The participant was instructed to stand in a dominantly upright position without applying contact force to neither shin nor calves. Here, the intention was to define a calibration procedure which can be easily transferred and performed under laboratory conditions as well as on the slope. For this calibration procedure no damped compensator (as presented in the research of Böhm and Senner, 2008) was utilized, as no significant differences were observed in the preliminary calibration tests of the force sensors. The calibrated values of the sensors revealed that the defined approach is reliable, consistent (cf. Table 2) and sufficiently accurate. Unwanted ski movements were not detected. After calibration, the sensor signals returned to baseline 0 N (of the Δ force) once the participant get to the calibration stand position.

**Table 2:** Calibrated sensor (tare) values of preliminary test runs [N].

**Run#**	**H**	**BL**	**BR**	**TR**	**TL**	**FR**	**FL**
**–**	**N**	**N**	**N**	**N**	**N**	**N**	**N**
1	−57.38	−6.80	−36.67	−5.11	−6.88	−88.67	−148.00
2	−57.33	−8.87	−33.28	−5.00	−6.91	−115.67	−216.87
3	−57.24	−8.40	−35.54	−4.84	−6.15	−136.70	−247.11
4	−57.24	−8.01	−28.44	−4.83	−6.27	−124.59	−205.38
5	−57.27	−8.73	−33.13	−4.88	−5.48	−126.85	−209.10
6	−57.28	−9.11	−54.28	−4.91	−6.28	−126.44	−214.23

Static pull positions where executed perpendicular to the ramp base position. The participant pulled slightly below shoulder height to maintain a natural skiing position. As the static center-weighted skiing position (standard position, SP) is concerned the measured knee angles resulted in mean flexion angles of 44 ± 10°^3^. Yoneyama et al. (2001) averaged at about 50° knee flex angle when comparing joint motion as well as reacting forces in the carving ski turn compared to a conventional ski turn. A similar ramp testing study (30° ramp covered with rubber) was conducted by Böhm and Senner (2008). Here, mean knee flexion angles of 45.8 ± 8.9° were determined.

### 2.3. Experiments on the Slope

The test runs where performed by a state certified Austrian ski/mountain guide and professional ski teacher (m = 75 kg; L = 178 cm). The usefulness of lightweight and simple systems in alpine skiing using IMUs^4^ as well as dGNSS^5^ sensors was underlined by recent studies of Gilgien et al. (2013), Gilgien et al. (2015), and Martínez Álvarez et al. (2019b). It was pertinent to combine IMU data with the non-intrusive characteristics of the lightweight force recording setup developed here. IMUs proofed to be useful to collect performance oriented skiing data from diverse body parts (Yu et al., 2016) to capture vibrations acting on the lower back using shank, thigh, sacrum, and sternum sensors (Spörri et al., 2017) and to aggregate data to accurately (±0.06 m) estimate the athlete's CoM (center of mass) (Fasel et al., 2016). The non-intrusive way of using IMUs is a highlighted advantage in skiing and other sports (Camomilla et al., 2018) as the package of the units is small and light, wearable, easy to set-up and to analyse. The latter comprises the translation of scientific data and expert evaluation into useful coaching hints for the user (Brunauer et al., 2020). Furthermore, the model of an inverse pendulum can be applied depending on the sensor position to interpolate CoM kinematics (Gilgien et al., 2013, 2015). Consistently, Martínez Álvarez et al. (2019a) have demonstrated, that their developed turn detection device is a valid and robust tool based on gyroscope sensors^6^. To that effect, the authors used aggregated raw data of Snowcookie^7^ IMU devices for the determination of position, speed and edging angles. Three IMUs were used, one positioned at the lower end of the sternum, the other two 5cm in front of the bindings' toepiece, resulting in additional 35 g each (55 g with mounting plate). The IMUs contain 9-axis motion sensors (gyroscope, accelerometer, magnetometer) and deliver data via Bluetooth data exchange standard (BLE 4.2).

## 3. Results

### 3.1. Tests Under Laboratory Conditions

#### 3.1.1. Flat Land Weight Transfer

The first—most simple—test started in neutral position without significant force on shins or calves, followed by forward and backward leanings. In order to validate the ski boot system, the participant repeated every procedure multiple times after taring the system again on flat ground. Leaning forward and leaning backward revealed clearly force patterns apart from the heel sensor (see **Figure 5**), which reacted while rocking up and down rather than forward and backward leaning. The *range of motion* (ROM) of the up and down rocking movement while laying back is illustrated in Figure 3A, in which a slight, almost inevitable counter-movement of the upper body is noticeable.

**Figure 4:**
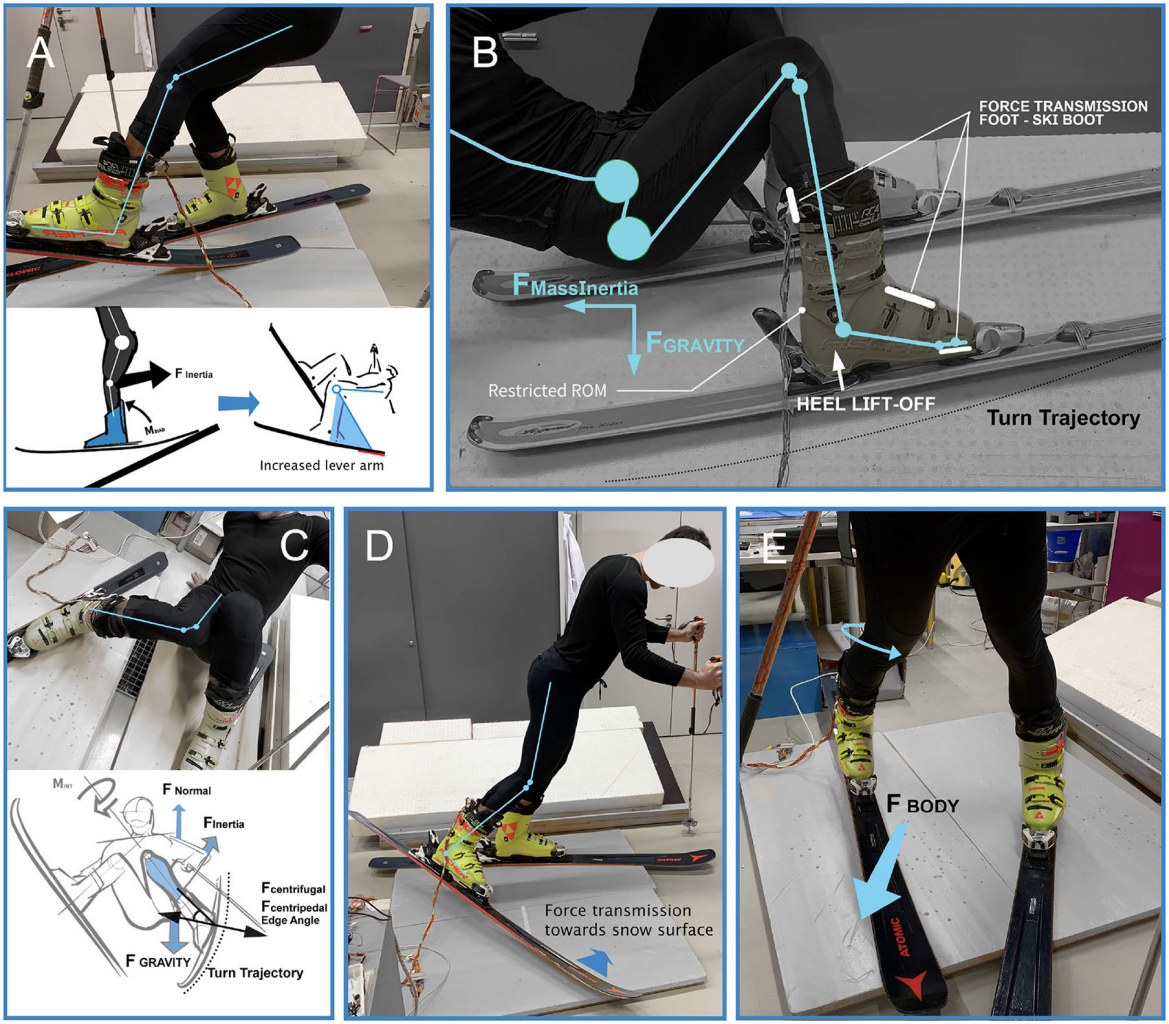
**(A)** Quasi-statical BIAD force appliance to boot; **(B)** Skiers' range of motion influencing the sensor force loading; **(C)** Phantom Foot: Increasing strain from a low starting position; **(D)** Inside edge pressure raise (cf. VER); **(E)** Slip-catch stance.

**Figure 5:**
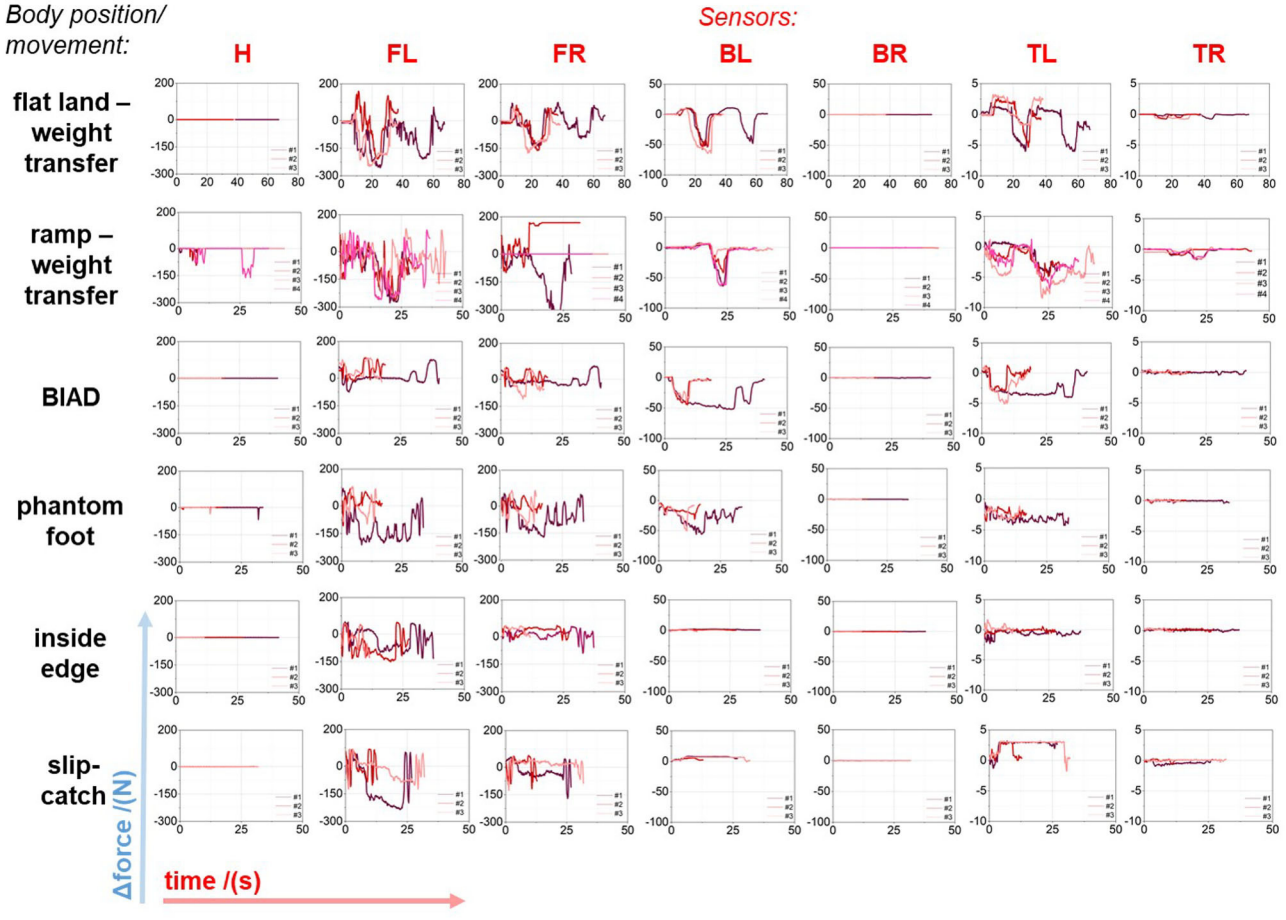
Referenced force over test time diagrams of all sensors. The force is referenced prior each measurement by a standardized procedure. Multiple measurement cycles are illustrated. Six skiing situations were simulated under laboratory conditions (body position and movement). BIAD, boot induced anterior drawer; inside edge …VER; slip-catch …hyperextension of the knee joint.

The flat land weight transfer sequence shows that forces are transmitted in the boots shell mainly due to the constrained ankle joint (cf. Figure 4B). Despite leaning backwards, the forces on the heel sensor hardly increase. This indicates limitations of solely in-plane force measurements. To overcome this limitations, force sensors have to be placed within the inner surface of the ski boot at the foot's and calf's contact areas. Additionally, the boot characteristics (e.g., ROM, boot stiffness represented by the *flex index*) influence the measured forces, significantly (Petrone et al., 2013; Hauser and Schaff, 2016). The ROM of the investigated ski boot is pictured in Figure 4B, where the participant is in a backward position with lifted heel and the right leg is rotated to the left-hand side. Thus, no force changes are detected at the heel sensor H and the right back spoiler sensor *BR* (see Figure 5).

#### 3.1.2. Ramp Weight Transfer

The same procedure, as previously described in the flat land weight transfer test, was conducted on the ramp. In addition, a lateral force was applied by pulling a dead-weight of an equivalent force of 336 ± 14 N. The back spoiler/shaft sensors (*BL, BR*) detected force changes while forward and backward leaning with respect to the calibrated body position, however, the heel sensor *H* only reached a peak force of 162 N when the participant was rocking up and down in neutral position while the pulling force was maintained (see Figure 5). The right upper toe sensor (*TR*) revealed small force deviations. The toe sensors showed strong force signals compared to the other body positions/movements, which is attributed to the direct force transmission in the forefoot area toward the ski.

#### 3.1.3. Quasi-Static Boot Induced Anterior Drawer (BIAD)^8^

Figure 4D illustrates how the effect of mass inertia of the skier and the increased lever from the back end of the ski are resulting in anterior tibia movement in relation to the femur while the musculus quadriceps femoris, the quadriceps ligaments and the ligaments of the patellae are aligned almost linearly and directly apply strain to the whole knee structure. During this test, the participant was instructed to push the back of the right ski downwards to the ground. The loading can be seen in the strong bending curvature of the ski (see Figure 4D). It is assumed that the quasi-statical BIAD position gives a first impression regarding the force distribution over the ski boot, although dynamic force peaks are neglected in this study. As seen in Figure 5, row 3, the back spoiler sensors (*BL, BR*) are indicators of the ski's back-end loading.

#### 3.1.4. Phantom Foot Position

From being in a severe but quasi-static valgus position of the knee joint (see Figure 4C), the loading was cautiously altered by the participant himself. Almost no pressure alteration is measured on the outside sensors/downhill side sensors. The most distinct signals come from the inside spoiler sensor *BL* which appear in combination with strong signals, partly exceeding the 200 N barrier, of the front sensors (*FL+FR*) (see Figure 5, row 4). Besides the participant felt a heavy loading in the inside-forefoot because of generating counter-pressure to hold the position under enough tension of the lower body. The force peaks of the heel sensor (*H*) could be induced by applying counter-pressure to fight the knee valgus movement, respectively to put even more pressure on the ski's rear edge.

#### 3.1.5. Inside Edge Pressure Raise and Slip-Catch Pose

The inside edge pressure raise position (*valgus-external rotation*) is shown in Figure 4D. In Figure 5 the toe-induced reaction of the front sole sensors (*FL+FR*) is observed as distinct peaks (great toe presses downwards and to the inside), whereas the heel sensor (*H*) remained unchanged. Low force changes of about 4 N are observed at the left spoiler sensor (*BL*) which is traced back to calf rotation inside the boot. Also the slip-catch pose (*hyperextension of the knee joint*) revealed comparable characteristics in terms of the force changes. The slip-catch pose is a similar loading as the inside edge pressure (cf. Figure 4E) besides the lifting of the ski. Therefore, the measured force changes are in the same range and show related force patterns.

#### 3.1.6. Conclusion Lab Tests

Regarding the constraining forces of a ski boot, the placement of the three insole sensors (two at forefoot region, one under the heel) should give a clear representation of the heavy fore-foot loads. Overall ground reaction forces are transferred through the boot's cuff and spoiler, hence, lead to differences compared to force plate results (Cf. Lüthi et al., 2005; Stricker et al., 2010). Nakazato et al. (2011) have shown that pressure insole measurements tend to underrate the force values from 21 to 54% depending on several factors (turn phase, skiing level, slope, etc.) and that different loading patterns occur over loading and unloading cycles of the ski turn.

Range of motion (ROM) restrictions in addition avoid clear force characteristics in the heel region apart in the static tests. As illustrated in Figure 4B, a substantial amount of force of the skier's lower limbs in the system^9^ is transferred across the upper spoiler and the instep areas into the ski boot's shell. The observation of merely pressure insole data becomes difficult to interpret in back-weighted positions, because of the force transmission in above mentioned areas. The spoiler forces and the rotation of the bootleg seem to be reliable indicators of the skiers' balance along the sagittal plane. Therefore, the development of a sensor-equipped hinge element that measures angular position, angular velocity and the resisting forces in the end positions (forward lean, backwards lean) could be of great interest for future studies.

On the contrary, the lab tests and the deduced results provided insights, that sensors in the upper toe area solely represent values of low forces (i.e., not exceeding ±5 N). Though force changes related to body movements on the sagittal plane are observed, which are influenced by the multiple joints in the forefoot and especially by arbitrary toe movements. Force change peaks especially occur when spontaneously changing body position or when initiating a ski turn. It is advisable to re-position the upper toecap sensors (*TL, TR*) to higher loading positions for further experiments. For example, the *cabrio* or *overlap* designs of a modern ski boot hardly allow sensor positioning directly at the instep and above the metatarsal bones. Further structural changes to the ski boot have to be reconsidered for experimentation in the future.

### 3.2. Testing on the Slopes

**Figure 6:**
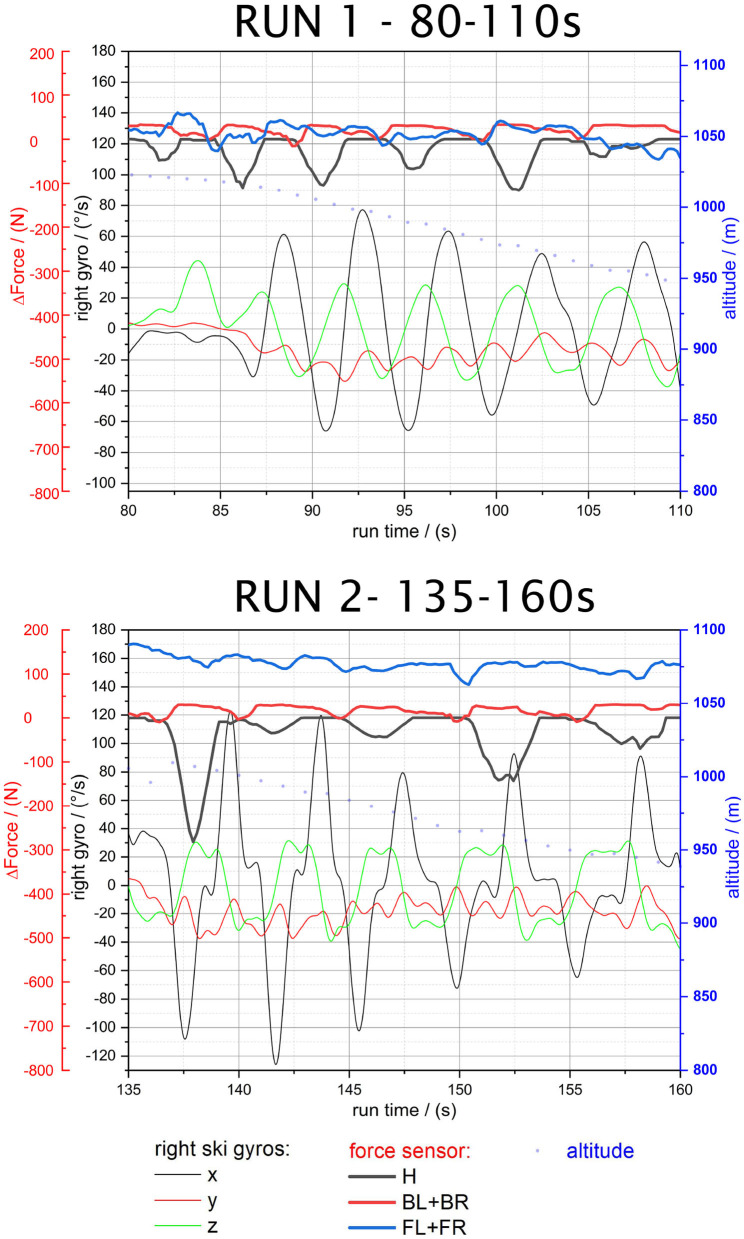
Run 1—80–110 s and Run 2—135–160 s.

**Figure 7:**
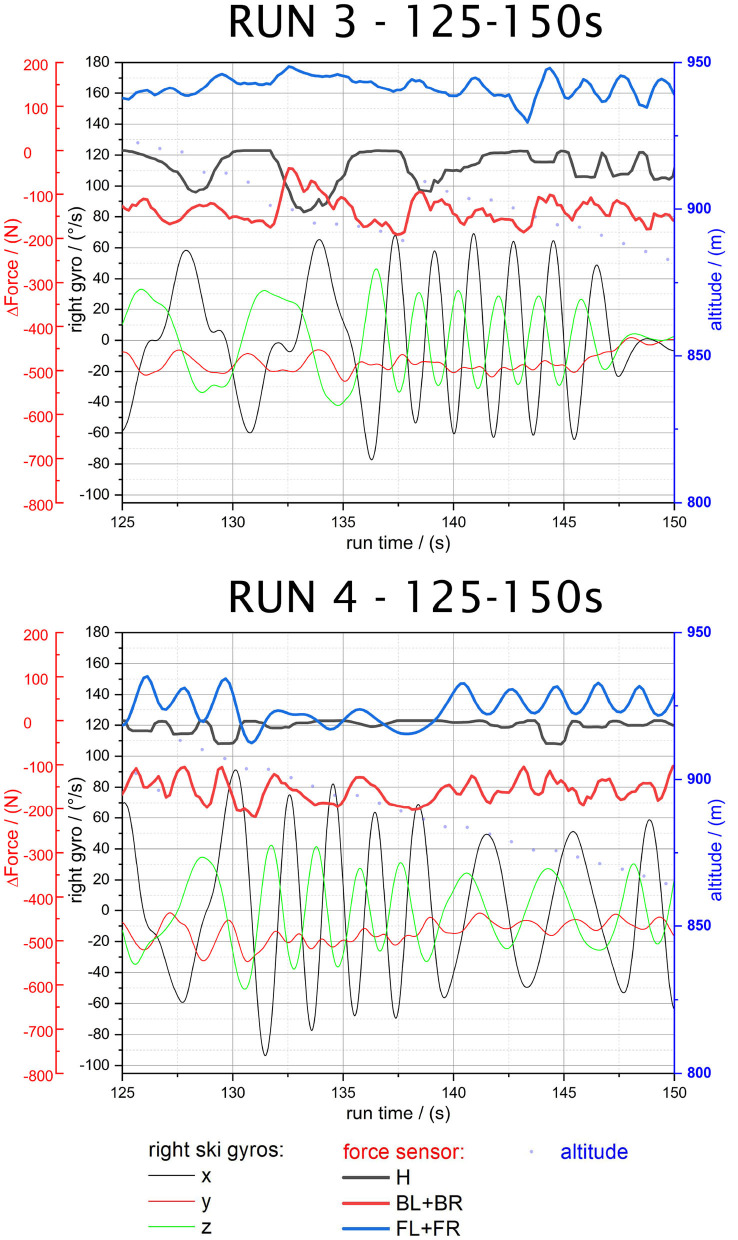
Run 3—125–150 s and Run 4—125–150 s.

**Figure 8:**
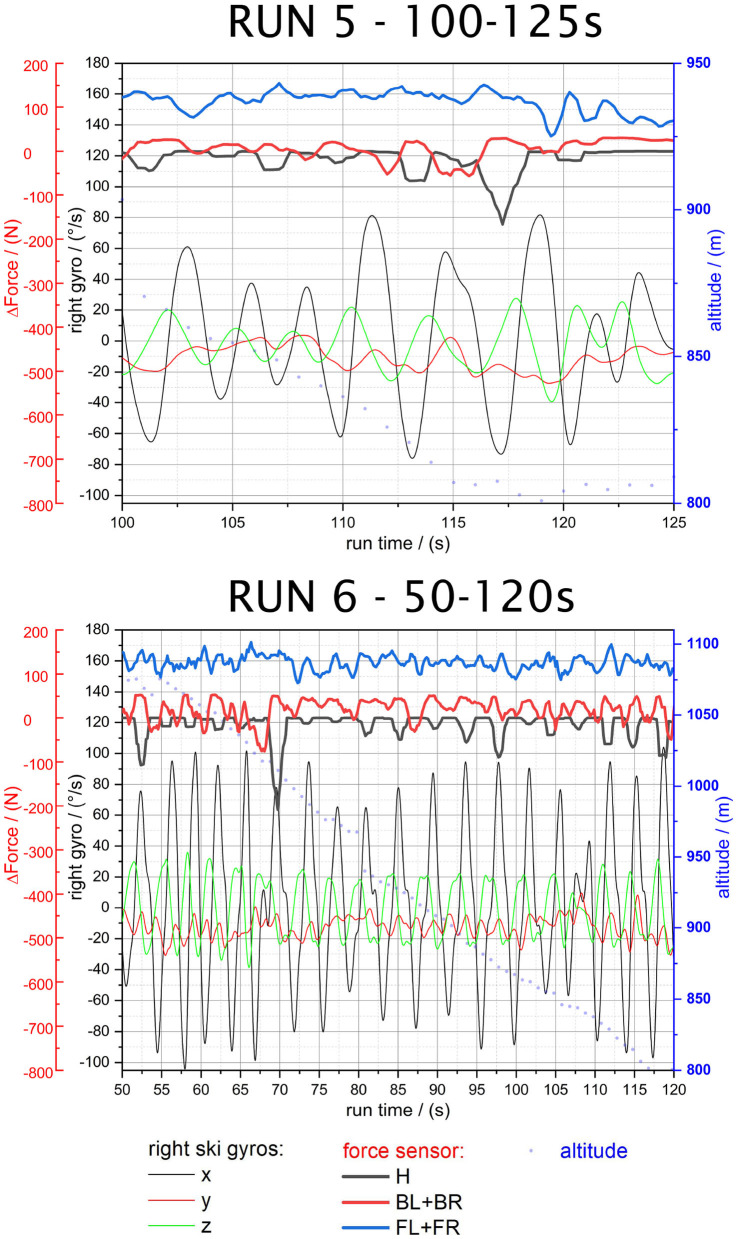
Run 5—125–150 s and Run 6—50–120 s.

The slope mainly has a steep incline of 25–35%. The edge inclination according to the Snowcookie data analysis resulted in 27° outside ski 40° inside ski for short turns in average (of the total number of short turns) and for longer turns 52° outside ski 56° inside ski were examined from the time integral of the gyro sensor data (see Figures 6–8; x-axis = axis along skiing direction/coronal plane, y-axis = steep incline of ski/sagittal plane, z-axis = rotation/transverse plane). The first two runs where performed at moderate speed alternating short turn and mid-to-long turn sections. Run **3** and **4** were performed with swing intervals increased forward lean and backward lean. Run **5** and **6** were performed at maximum speed with regard to the moderate steepness of the slope. Table 3 lists the measured velocities (maximum and average) during riding on the slope including gliding passages of the slope. The data recording paused when the skier stopped and so were not included in the evaluated data (Snowcookie data were used to reference all recorded data).

**Table 3:** Maximum and average velocity of each run on the slope in [km/h].

**Run#**	**Max. velocity**	**Average velocity**
**–**	**km/h**	**km/h**
1	59.9	34.2
2	58.9	36.1
3	50.9	36.2
4	55.2	36.1
5	87.1	53.0
6	74.7	53.1

**Figure 9:**
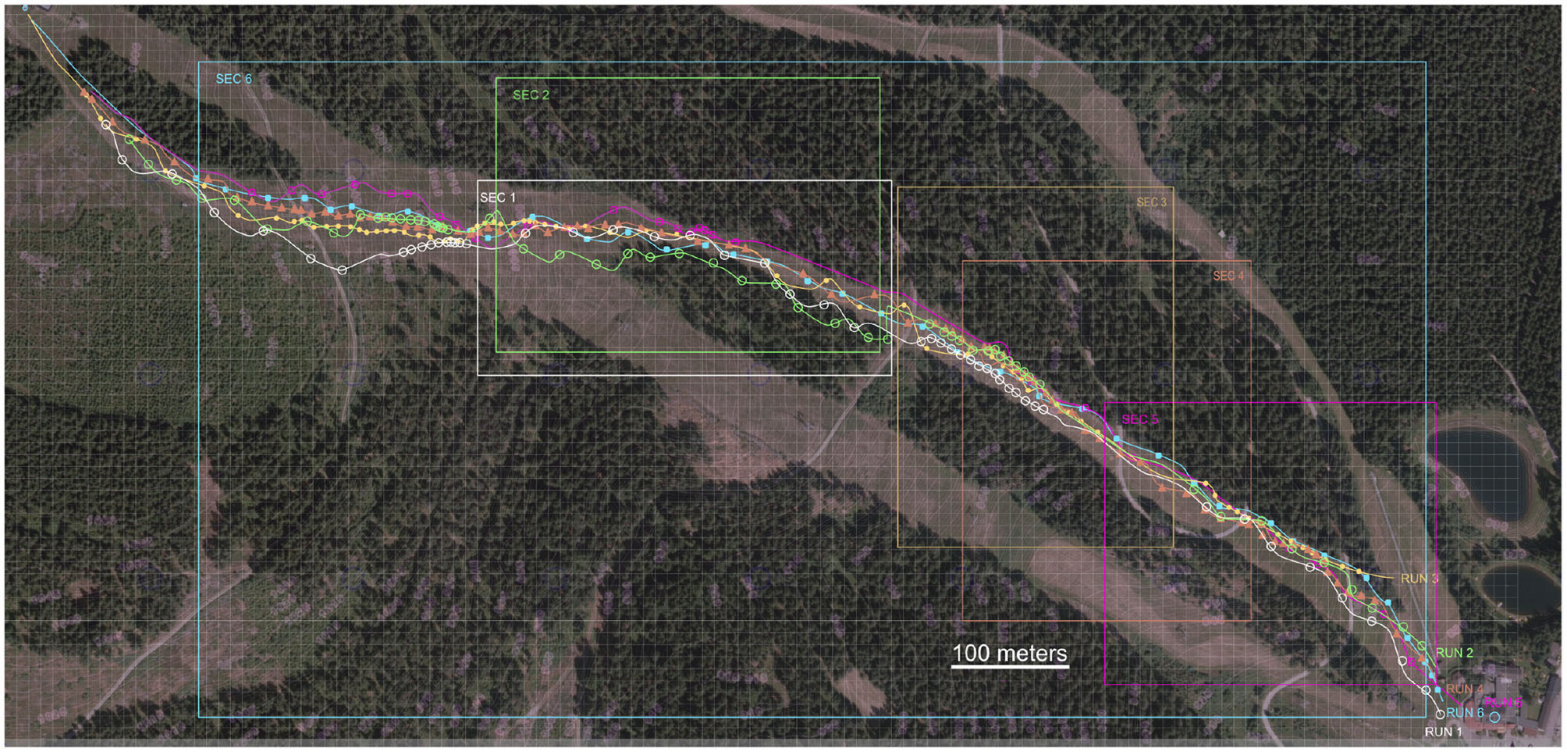
Overview of six runs—each section marks the range of the detailed run analysis as shown in the diagrams from Figures 6–8.

**Figure 10:**
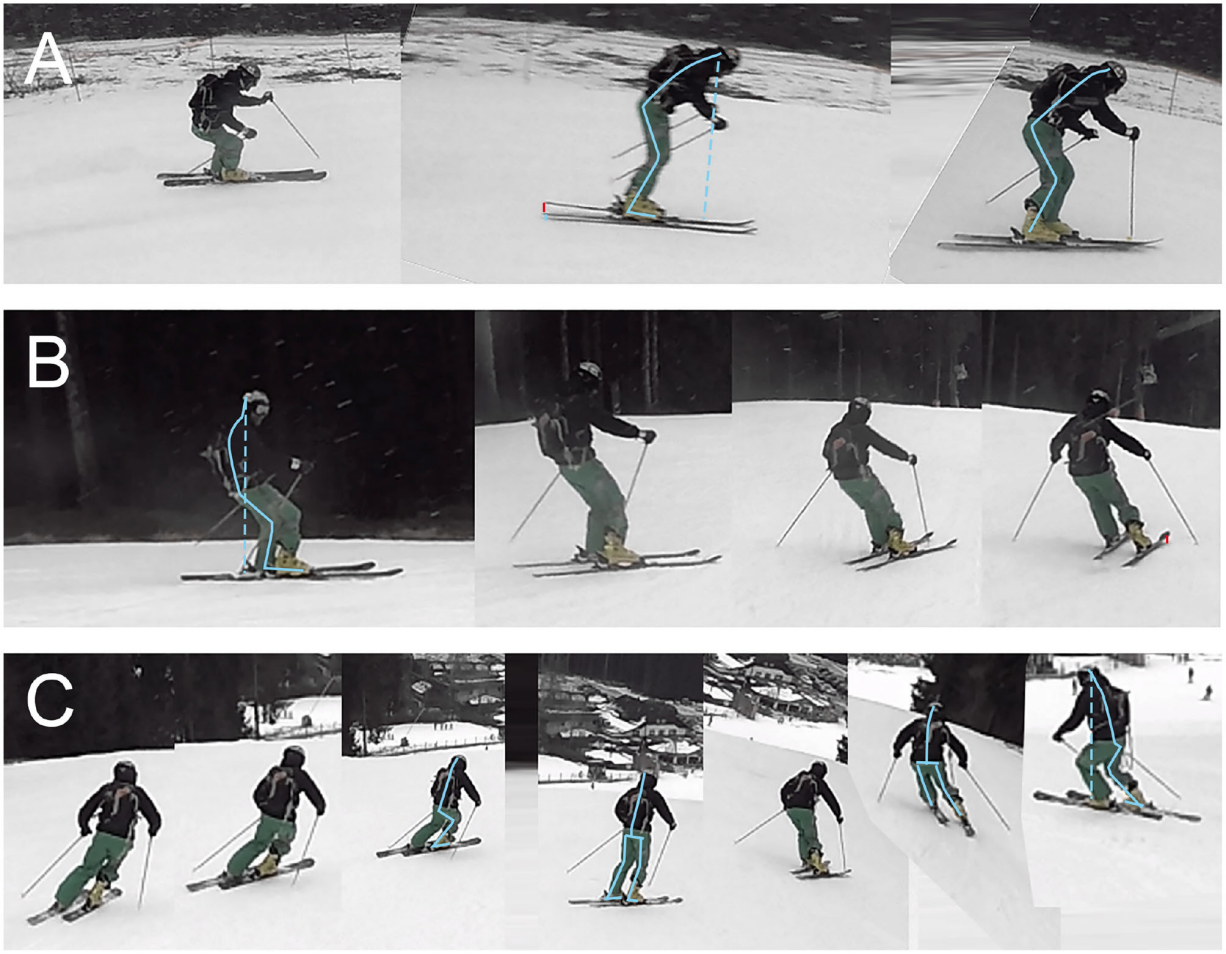
Still frames from video footage: **(A)** Forward lean skiing. Strong upper body overhang with overdone steering initiation via the ski's shovel **(B)** Lay-back positions with center of mass clearly behind the foot area. Upper movement necessary to initiate a turn. No distinct hip bend and no clear longitudinal foot separation (skiing direction). Nonetheless, a slight forward lean during the turn transition was necessary. **(C)** Balanced skiing with clear hip bend and compact body position. The body extends during the turn transition and unloading of the skis.

All runs were executed with alternating phases of long turns and short turns. As the tests were done on public slopes and warm conditions (+2° C), the rider had to adjust his individual lines as illustrated in Figure 9. Furthermore, this figure shows the sections the authors picked from all six runs to showcase detailed views. Figure 10 showcases still footage of forward lean, lay-back and centered skiing as described below:

The results of the recorded force changes are shown for different runs and sections (see Figure 9). Taring results and overall forces during skiing indicate that the sensors on the insole and the spoiler are reliable and sufficiently accurate for this descriptive study. DAQ resolution and frequency (cf. section 2) are adjusted in order to detect turns and balance parameters. The force sensor data were correlated to the Snowcookie data and analyzed in the following. In the following diagrams, the run-time dependent change of the (calibrated) Δforce of the sensors TL and TR are omitted as they confirm the results of the laboratory ramp test and reveal low measured forces. In addition, the measured data are highly scattered, mostly, leading to non-conclusive patterns. The back spoiler sensors (*BL, BR*) were combined as differences in the calf shapes, the individual knee position, respectively the boot's canting^10^ seemed to influence the loading contribution strongly in this setup. The insole sensors (*FL, FR, H*) revealed clear force changes and demonstrated the strong influence of the toes, respectively the forefoot.

#### 3.2.1. RUN 1 and RUN 2: Regular Skiing: Long Turns and Short Turns Intervals

The spoiler sensors (*BL+BR*) demonstrate that there are slight forces changes in a range of 50 N (see bold red line in Figure 3). Force peaks are reached close to the highest inclinations of the x-axis (ski direction) (black thin line) of the right turn (inside boot) already accelerating the ski toward a left turn. Interestingly, the toe sensors (*TL+TR*) reach their force peaks just before the heel sensor which can be associated with a central balanced stance on the skis and the turn initiation by transferring force and inclination toward the front edges of the skis. Because of the neutral stance while taring, the back sensors even show slightly positive values (less force, unloading) at the swing changes and the beginning of the turns.

#### 3.2.2. RUN 3 and RUN 4: Forward Lean Skiing and Skiing With Weight Backwards

The third and fourth runs were executed alternating with extensive forward lean as well as with backwards shifted weight. The sole sensors (*H, FL+FR*) revealed no significant force deviations, however the force patterns from run 3 and 4 are different as the timing of the swing release is varied and the ski acceleration differs. The sole sensors are depending on edging angle and acceleration forces while the back spoiler sensors (*BL+BR*) relate more directly to body position. The back spoiler sensors clearly show deviating force peaks, partly exceeding forces of 200 N, as seen in the back-weighted skiing sections of Figure 7. This is of particular interest for further studies, where detailed correlations of actual body position to force change patterns have to be drawn.

#### 3.2.3. RUN 5 and RUN 6: Fast Skiing

Run 5 and 6 were performed at higher speeds and a more radical deceleration on the transition from long turns to the short turn phase. Overall, the steep incline of the slope was moderate and more edge force would have led to deceleration and despite that, the force peaks on the insole positioned heel sensor (*H*) reveal significantly higher force changes as expected. For faster skiing the weight transfer from (*FL+FR*) toward (*H*) at the end of the turns becomes clearer.

#### 3.2.4. Conclusion Slope Tests

##### 3.2.4.1. *Toe induced turn release*

The release of the short swing turns as well as the long radius turns is often indicated by a quick force increase of the sensors *FL* and *FR* and the direction change is linked to a general reduction of ground reaction forces (*FL, FR, H*).

##### 3.2.4.2. *Backward lean*

Backward lean has to be considered in relation to the slope inclination and becomes obvious during a loading phase of the ski turn (*BL, BR*). Further sensor units positioned on the instep and directly at the hinge could give further informative data about the body position. Therefore, the overall structure of an instrumented (smart) ski boot for further testing has to be optimized.

##### 3.2.4.3. *Less force peaks on heel as initially expected*

Due to the strong form fitting of a modern ski boot, the heel gets levered upwards because of the direct contact of the instep and the restricted ROM influenced by many parameters, such as cuff height, cuff geometry, hinge point, material properties, temperature, among others. In that regard, torque measurement in the boot's hinge, comparable to bicycle power-meters, could give more information about the boot's stress loads and deformation.

## 4. Discussion and Outlook

The aim of this study was to assess piezoresistive sensors and their positioning (Figure 1) within the ski boot to study whether or not force changes as well as patterns are observable regarding skiing balance, riding style or severe (injury related) body positions. The sensor positions are of particular interest for a future injection-molded boot design produced by inserting the sensors directly into the mold prior to the polymer injection. Additionally, the positions were chosen to gain insights of injury related movement patterns. Our results reveal that force patterns for specific body positions as well as movements can be derived and regarding the positioning of the sensors the conclusion is that the toecap (upper foot; *TL, TR*) sensors are not essential to derive force patterns at their current position. This was examined under laboratory conditions as well as on the slope. A positioning further toward the instep, respectively above the metatarsal bones, will lead to more consistent results. Consequently, the noise of the data by arbitrary toe movements while skiing will be reduced. For athletes and, especially, young athletes, the back spoiler sensors (*BL, BR*) and the toe sensors (front sole; *FL, FR*) in combination with the data of accelerometer and GPS can highlight athletes the efficiency and more importantly the force acting on the ski boots toward the end of a turn when acceleration by subtle weight transfer is desired. Especially, accurate location tracking technology already provides valid data about ground reaction force (GRF), air drag and snow friction in ski racing (Gilgien et al., 2018) and might help the analysis of force data in future in regards of external forces and risk evaluation. Moreover, it is necessary to measure a wide force range with calibrated piezoresistive sensors rather than only the distribution of the skiers weight (without considering inertia effects). This approach is a further development of pressure insoles as well as devices that measure body position (some commercial products are recently available^11^). As those devices actually measure weight distribution over a sensor map, algorithms can help skiers to gain deep insights about their skiing, immediately.

Further research has to be conducted to analyze the presented ski boot with sensors in terms of limitations, temperature dependent force changes due to the inherent viscoelasticity (i.e., loading rate, humidity and temperature dependent behavior) of the boot material. To that effect, the influence by the strong form-fitting of the stiff ski boots shows significant influence on the measured forces (force paths) and has to be studied in detail by optical full-field strain analysis under loading. Beyond this qualitative study, a further matching with optical systems that are capable of capturing motion in 3D space (Federolf, 2005; Spörri et al., 2016; Rhodin et al., 2018) has to be analyzed in future studies to render more accurate bio-mechanical conclusions.

Furthermore, this study has to be extended by increasing the participants (skiers) with variations of skiing level, style, age and gender in order to investigate representative results statistically (hypothesis test for bias and Pearson's correlation coefficient). One challenge for this representative study will be the preparation of ski boots in different size. The next step would include a feasibility study for a manufacturing process, such as injection molding technology, that enables the sensors to be safely molded within the ski boots' shell while being protected by a suitable material matrix (cover). This would also involve the testing of fatigue phenomenons of sensors, boot materials and the analyses of the boot stiffness in comparison with the common ski boot model. A new design of a ski boot would be necessary to find an optimized purpose-driven form for measuring skier related forces in the boot.

Future findings should show more clearly how ski binding release could work, as there are many options to integrate electro-mechanical release systems into the ski-binding-boot-system (SBB). A holistic understanding of skiing safety is therefore required. With this, a predictive (AI-algorithms based) binding system can be implemented in an electro-mechanical binding. The authors are convinced that this can contribute to rethink current standards of alpine safety products. Skier, basically, have a lot of trust to the reliance of state-of-the-art mechanical bindings. Also it seems that skiers have a greater awareness of anterior cruciate ligament (ACL) injuries. That is why the combination of a basic mechanical solution and an additional electro-mechanical release device could be an intermediate step to gain the customer's trust for smart electro-mechanical (IoT) release solutions. Additionally, the development of a smart cuff-hinge has to be considered in future for several reasons: (i) a sensor at the hinge can monitor forces as well as torques during cuff rotation (tibia position); (ii) a sensor-equipped cuff hinge will be independent of boot size and boot model, consequently, sensor data can be interchanged; (iii) there is already proven technology commercially available for other sports (e.g., power measurement in the crank axle of bicycles or oarlock power-meters in rowing). As the authors investigate ski binding release action (Nimmervoll et al., 2021) and the potential of a binding plate with lateral release functionality, this work already delivers important data as a preparatory study. Predictive data analysis can give the skier feedback about the release settings. The skier can see how close a situation came toward release in regards of occurring forces and what happened when, for example, accidental release happened considering as a false positive. This can lead to individual release profiles also adjustable to the utilization of the ski equipment (e.g., park, back-country, slope, race, couloirs). By utilizing an electro-mechanical binding plate with an adjusted retention force, lateral release in certain situations can be unlocked and locked within milliseconds without sacrificing the qualities of state-of-the-art mechanical bindings. Binding elasticity^12^ is a crucial attribute of mechanical bindings to avoid false positive events and can save the skier from dangerous falls. Safety ski bindings are designed to release during the forward fall (heel piece releases upwards) and during a forward fall with body rotation (toe piece releases side-wards). Regarding this basic functionality, bindings are more unlikely to release in backward-twisting falling situations. The assumption is, that a laterally releasing binding plate can assist a mechanical binding to the overall release forces in certain situations dependent on the real-time measurement of certain in-boot sensors.

The potential of measuring forces inside the ski-binding-boot-system can be considered on several levels and always in combination with other environmental data (e.g., steep incline, body position, force acting on the ski boots). This study contributes to the visionary idea of measuring the skiing speed and acceleration (dGNSSR/GPS, 6-axes DOF accelerometer) in real-time and triggering, beyond an electro-mechanical ski release binding, the following: (i) helmet airbags and stiffening joint protector to restrict the body's range of motion during falling; (ii) high loads with severe backwards or forwards lean can trigger, among other signals, a mechanism for lateral binding release and adaptively reduce the release force values. Furthermore, smart bandages/protectors can be utilized to stiffen vulnerable body areas (e.g., knee joint) and restrict the ROM to avoid hyperextension and hyperflexion when overloads are detected. These and other developments of ski bindings, ski boots, and other wearable equipment will help to increase the safety of skiers and provide force pattern analyses of turn initiation and acceleration, which is particularly beneficial for the development of skiing technique for (young) athletes.

## Data Availability Statement

The original contributions presented in the study are included in the article/Supplementary Material, further inquiries can be directed to the corresponding author/s.

## Ethics Statement

Ethical review and approval was not required for the study on human participants in accordance with the local legislation and institutional requirements. Written informed consent was obtained from the individuals Florian Nimmervoll, Umut Çakmak, and Alwin Mold for the publication of any potentially identifiable images or data included in this article.

## Author Contributions

FN and UÇ contributed to the conceptualization of the study, the prototyping, the testing, and the analysis. MR contributed to the prototyping. All authors contributed to the article and approved the submitted version.

## Conflict of Interest

The authors declare that the research was conducted in the absence of any commercial or financial relationships that could be construed as a potential conflict of interest.

## Footnotes

^1^For example, software platforms like *Strava* or *Zwift* in the endurance sports sector.

^2^cf. https://www.academia.edu/7865995/Mechanism_of_ACL_Injury_in_Skiers_Letter_to_the_Editor.

^3^Measurements were performed with a protractor, thus, accuracy is lower compared to an optical 3D tracking system using visual pointer markers.

^4^Inertial Measurement Units.

^5^Differential Global Navigation Satellite System.

^6^LSM6DS3, (2.5 × 3 × 0.83) mm, ±8 g, and ±500 dps full scale, STMicroelectonics, Amsterdam, Netherlands.

^7^https://snowcookiesports.com/startup-home-2/.

^8^Boot-induced anterior drawer is a mechanism caused by a backward fall applying severe stress on the knee structures as a consequence of the skier's inertia and the stiff boot structure (cf. Johnson et al., 1983).

^9^Skier—boot—binding—ski—ground.

^10^The cuff canting adjustment of the lateral tilt enables the skier to tune the boot geometry to the specific leg morphology.

^11^For winter season 2019/20 a product called *Carv* was released. https://getcarv.com/app.

^12^Travel of the binding before it releases the boot.

## Supplementary Material

Supplementary Video 1 Lab test-weight transfer and taring position.mp4.

Supplementary Video 2 Slope test.
